# A Qualitative Study to Assess US Patient Preferences between new Transdermal System and Injectable Anabolic Therapies for Osteoporosis Treatment

**DOI:** 10.1007/s11657-022-01075-z

**Published:** 2022-04-04

**Authors:** Charlotte Beaudart, Stuart Silverman, Deborah T. Gold, Setareh A. Williams, Rich Weiss, Mickael Hiligsmann

**Affiliations:** 1 Department of Health Services Research, CAPHRI Care and Public Health Research Institute, Maastricht University, Duboisdomein 30, 6229 GT Maastricht, the Netherlands; 2 Cedars-Sinai, Los Angeles, CA USA; 3 University of California Los Angeles Medical Center, University of California Los Angeles, Los Angeles, CA USA; 4 Department of Psychiatry & Behavioral Sciences, Duke University Medical Center, Durham, NC USA; 5 Radius Health, Inc., Boston, MA USA

**Keywords:** osteoporosis, stated preference, focus groups, qualitative research, anabolic treatment, fracture

## Abstract

**Mini abstract:**

US patients with osteoporosis included in three focus groups identified efficacy, safety, cost, and convenience as important attributes of treatment when choosing between anabolic therapies with high stated preference for the solid Microstructured Transdermal System.

**Objective:**

The current study evaluated patient perspective and relative importance of treatment attributes of in-home daily self-administration of abaloparatide-solid Microstructured Transdermal System (sMTS) compared with other anabolic agents (i.e. in-home daily subcutaneous self-injections, and monthly subcutaneous injections at doctor office) among a group of US patients with osteoporosis.

**Methods:**

The current study included systematic literature reviews, experts’ consultation and three online patients focus groups (n=27), including patients ≥50 years of age at high risk for fracture. Nominal Group Technique was used by asking patients to (1) Individually identify characteristics that would be important for them when choosing between anabolic treatments, (2) Share ideas and discuss perspectives with other patients, (3) Review additional attributes generated from a systematic literature review, (4) Select and rank individually the 7 most important characteristics from the list and (5) Report their acceptability and stated preference ranking between the three treatment options.

**Results:**

Twenty women and 7 men with a mean age of 65 (range 51-85 years) participated in the focus groups. Twenty-four treatment characteristics were identified through focus groups and literature review. Efficacy, safety, out-of-pocket costs, strength of evidence and the option to self-administer were ranked as the most important attributes. The majority of patients stated preference for a daily sMTS if prescribed by their doctor.

**Conclusions:**

This study revealed that efficacy, safety, costs, and convenience are important attributes of osteoporosis treatment for US patients at high risk for fractures when choosing between anabolic therapies, with a high stated preference for sMTS.

## Introduction

Osteoporotic (OP) fractures result in significant morbidity, excess mortality, functional decline and decrement in health-related quality of life [1, 2]. OP fracture incidence, which was decreasing previously, is no longer declining and may be on the rise, an observation that parallels a decline in screening and treatment initiation [3, 4]. High discontinuation rates following treatment initiation including suboptimal adherence with OP medications remain a problem in disease management [5, 6] and are associated with increased clinical and economic burden [7, 8].

The increasing humanistic and economic burden of osteoporosis in the US, including suboptimal adherence [9, 10], suggests the need for additional treatment options, especially in high-risk populations. Patients’ treatment decisions are dependent on their expectations and acceptability of treatment. Osteoanabolic treatments, which are indicated for patients at high risk for fractures, are only available as subcutaneous injections. According to a recent survey conducted by the National Osteoporosis Foundation, some patients at high risk for fracture are not willing to take medication because it is only available as an injectable [11]. Radius Health, Inc., in collaboration with Kindeva Drug Delivery L.P., is developing a drug-device combination product for a transdermal method of abaloparatide administration, the abaloparatide-solid Microstructured Transdermal System (abaloparatide-sMTS) (WearABLe study, NCT04064411), which consists of a small polymeric disk of microneedle arrays coated with abaloparatide [12]. This new route of administration may be an alternative treatment option for patients who do not find conventional injectable therapies acceptable. According to the phase Ib usability study (NCT04366726, n=22), the daily administration of abaloparatide- sMTS resulted in consistent PK profile and increases in bone turnover markers similar to those observed in the ACTIVE phase 3 study with the abaloparatide subcutaneous administration and was associated with non-detectable pain in most patients [13].

Understanding patient perspective is of paramount importance in identifying the best treatment option for the individual patient. Furthermore, characterization of patients who would be more accepting of a particular mode of intake can inform shared decision making between the prescriber and patient. The objective of the current study was to evaluate US osteoporosis patient stated preference and relative importance of treatment characteristics of abaloparatide-sMTS compared with currently approved anabolic therapies including daily and monthly SC-injections.

## Methods

The study design was consistent with the FDA guidance for conduct of patient preference research [14, 15]. The protocol (Radius Health, Inc.-HEOR-006) was developed and approved by the research team including two experts in preference research, two US osteoporosis experts with experience in preference research, one endocrinologist, one chronic disease epidemiologist, and one osteoporosis patient ambassador. A two-step research approach involved a systematic literature review, including a consultation with subject matter experts and a patient ambassador/advocate, and three focus groups with US osteoporosis patients.

### Selection of attributes for the focus groups

Two systematic literature reviews were carried out following the Preferred Reporting Items for Systematic Reviews and Meta-analysis (PRISMA) statement throughout the whole procedure [16]. The first systematic review included patient surveys, conjoint analyses and reviews about patient’s preference focusing on the identification and determination of osteoporosis treatment characteristics of importance to patients. The second systematic review included a review of preference/satisfaction studies for transdermal patch and mode of administration in all disease areas. Medline and Cochrane Central Register of Controlled Trials (CENTRAL) (via Ovid) were searched in August 2020 using two search strategies available in Appendix A. The search was limited to papers published in English [17] excluding letters, editorials, and case reports. In addition to the searches on bibliographic databases, a hand search of references of included studies or relevant papers in the field was also performed. Furthermore, a manual search for guidelines, regulatory recommendations or advocacy group perspectives was also conducted. To supplement the literature review, insights were also ascertained from primary healthcare providers during an advisory board meeting regarding their perspective of patient preference for osteoporosis treatment attributes. Lastly, a discussion with a patient ambassador was carried out to identify any additional attributes that were not identified through the above approaches.

The decision of keeping or removing the attributes identified from the literature review for focus groups was performed in consensus with the research team. All attributes were displayed as a list and each member of the research team was asked to select only the attributes they identified as relevant for the focus groups. To be included in the focus groups, treatment attributes needed 1) to be relevant for osteoporosis, 2) differentiate between sMTS and SC-injections, 3) be conceptually different from each other, 4) be relevant to the objectives of the study.

### Focus groups

Three online focus groups with US patients were conducted to understand key treatment attributes of importance to patients in their choice between abaloparatide-sMTS and daily or monthly SC-injections.

#### Participants

Patients were considered eligible to participate if they qualified for anabolic therapies including age ≥50 years and at high risk for fracture defined as having a history of osteoporotic fracture, multiple risk factors for fracture, or treatment failure or intolerance of other available osteoporosis therapy. External recruitment was made by the Contract Research Organization (CRO) Global Health Perspectives using ad hoc recruitment network including local experts’ database, Healthcare Professionals (HCP) referrals, social media, and advocacy groups. Participants were thus not aware of the industry involved in the research and did not know anybody prior of the study, neither the experts involved in the study, nor the moderator. Since this was a stated preference study and not an observed preference evaluation, participants did not need to have any experience with the treatment options for being included in the study.

During recruitment and in the information sheet as well, participants were informed that we recruited patients with osteoporosis or being at high risk of fracture. Efforts were made to recruit patients representing the full spectrum of osteoporosis patients at high risk for fracture including diverse geographical location (at least one third from each urban and rural setting), demographic (at least one quarter male, one third 50-65 years of age and one third 65+ years of age) and clinical (at least one third with/without a prior osteoporosis fracture, one third with/without prior osteoporosis treatment history) characteristics.

Each participant signed an informed consent form (ICF). The protocol and supporting documentations were reviewed and approved by Advarra IRB (Pro-00045119-Single-Site Protocol; August 12, 2020). The study was Health Insurance Portability and Accountability Act (HIPAA) compliant. Participants received a financial compensation for their time investment. The compensation amount was based on fair market value and approved by Radius legal and compliance team in addition to the independent Institutional Review Board.

#### Data collection

Participants with a signed ICF completed an online questionnaire prior to focus group participation, which included assessment of demographic and clinical history, using *Qualtrics* survey™ (full version of the survey is available in Appendix B). Subsequently, three in depth online focus groups were conducted using *Go To Meeting* platform to evaluate and prioritize the key treatment characteristics when choosing between abaloparatide-sMTS, daily and monthly SC-injections. The research team conducted a mock trial to ensure the appropriate construct of the online questionnaire and to test functionality of the platform. *Go to Meeting* platform allowed individuals to contact the moderator directly and separately from other study participants, to provide responses to the moderator questions and to have audio/visual access to the materials being discussed including video and slide illustrations. Family members were not allowed to help with the interpretation of questions during the focus group discussion or provide input or responses on behalf of the patient.

**Fig. 1.**
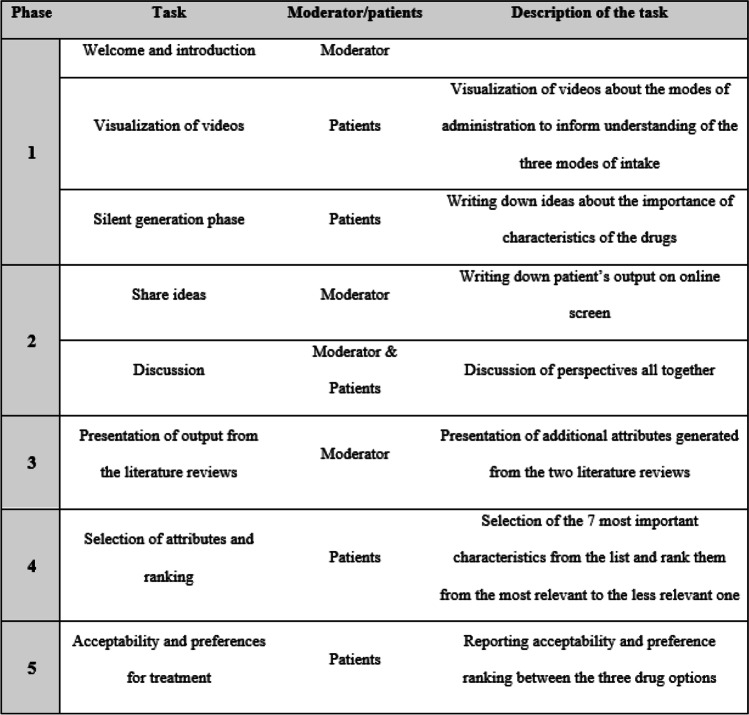
Flow diagram of the focus groups

Focus groups were conducted using the Nominal Group Technique (NGT)[12], which allows for inclusion of all participants’ perspective and therefore suitable for the identification and prioritization of treatment attributes to choose osteoporosis treatment [18–20]. Structured interview guide for focus groups was used. The methodology employed for the focus groups is summarized in Fig. 1. Each focus group started with an introduction of the moderator, the project, and each participant. Since this was a stated preference study, in order to assess participants’ perspective of available anabolic treatment options, videos demonstrating administration of each therapy (i.e., daily sMTS application, daily SC-injection, and monthly SC-injection, with no mention of the name of the drugs) were presented. Each video lasted about 1 minute and included background information about the mode of administration while providing explanation and time in a comparable way for the use of the 3 products. It was stated in the videos that sMTS and daily SC-injection can be self-administered at home and that monthly SC-injection requires a monthly visit to the doctor. Pain associated with administration was not mentioned in the videos. The videos were developed by the researchers using the products in their available form at the time of study execution and included the product in development.

After watching the videos, patients were asked to: (1) Individually write down ideas about the importance of various characteristics for osteoporosis treatments presented in the videos (i.e. silent generation phase), (2) Share ideas (written down by the moderator on online screen) and discuss perspectives with other patients, (3) Review additional attributes generated from the two systematic literature search presented as list by the moderator, (4) Select individually the 7 most important characteristics from the list of attributes compiled and to rank them and (5) Report their acceptability and stated preference ranking between the three products.

After each of the first two focus groups, a debrief was held with a 1-2 patient volunteers to ensure questions were clearly understood during the session. After each focus group, transcripts were also reviewed by the research team. Improvements were made to further clarify presentation of materials and facilitate discussion in subsequent focus groups accordingly.

#### Data Analysis

Interviews and focus groups were audio-recorded, then transcribed verbatim and thematically analyzed. The anonymity of the respondents was ensured in the transcriptions. Data analysis began after each focus groups: the researchers controlled for topic saturation during the next focus groups. Handwritten notes were also captured and analyzed afterwards. Themes were derived from the systematic literature review previously performed and considered in the context of focus groups analysis. The analysis and the coding were conducted on hand, by the first and last authors without using any computer software. The coding was finally discussed between all researchers to ensure the validity and credibility of the results.

Socio-demographic and medical characteristics of the participants were analyzed descriptively using frequencies (absolute and relative) for binary and qualitative variables and median (interquartile range) as well as minimum-maximum values for quantitative variables. The normality of distribution of continuous variables were checked using the Shapiro–Wilk test, histograms, Q-Q plots and the distance between mean and median [21].

Characteristics generated through literature reviews and focus groups were ranked individually by the six research members regarding their inclusion and importance, and then discussed/approved collectively. They were further consensually categorized by the research team into four domains: Efficacy, Safety, Cost and Convenience. The frequency with which participants included each attribute in their top 7 most important attribute was calculated. Based on the mean importance score and calculated frequencies, a ranking of characteristics was made from most to least important. Exploratory subgroups analyses were also performed. Subgroups were defined by socio-demographic and clinical characteristics (i.e., age, sex, very high risk of fracture defined by the following condition: having a previous hip or vertebral fracture or fracturing a bone during the last year or having at least two previous fractures).

The acceptability and stated preference for daily sMTS application vs SC-injections expressed in percentages were also calculated, first for the whole population, and subsequently by pre-specified subgroups of interest (i.e., age, sex, history of fractures). All statistics were performed using SPSS Statistics 24 (IBM Corporation, Armonk, NY, USA).

## Results

### Literature reviews

The PRISMA flowcharts for literature reviews are available in Appendix C. The first literature search, focusing on osteoporosis treatment, included 37 individual studies providing a list of 30 attributes. The second literature review, focusing on transdermal patch in all disease areas, included 37 individual studies providing an additional list of 21 attributes.

After review of attributes by the research team, 15 of the 51 identified attributes were considered relevant and were selected for presentation to the focus groups.

### Focus groups

#### Patient Characteristics

Of 30 patients invited to participate in the study, two were unable to connect to the internet, and one joined the online meeting but could not be heard. A total of 27 patients participated and were included in three focus groups according to their date of inclusion (focus group 1 [n=9]; focus group 2 [n=8]; focus groups 3 [n=10]) including 20 women (74.1%) with a mean (min-max) age of 65 years (51-85). The majority of the participants were non-Hispanic white (85.2%), were taking osteoporosis medication at the time of the focus group (81.4%) and had a prior fracture (59.3%). More than half (59.3%) had experience taking or giving an injectable medication (either subcutaneous or intravascular injections) and a third used self-injectables themselves. Twelve patients reported having previous experience with osteoporosis medication administered through subcutaneous injection. Four patients reported having received an intravenous administration of their osteoporosis medication. None reported having a fear of needles that would prohibit them from taking injectable medications. The characteristics of participants are presented in Table 1. Mean time of duration of focus groups was 104 minutes (95 minutes for FG1, 109 minutes for FG2 and FG3).

**Table 1:** Characteristics of study participants

	All (n=27)		All (n=27)
**General socio-demographic and medical characteristics**			
Sex (n, %) Women	20 (74.1)	Age (years) Min-max Median (P25-P75)	51-85 65.0 (59.0-69.0)
Race (n, %) Non-Hispanic White Non-Hispanic Black/African Amer Hispanic Non-Hispanic Asian	23 (85.2) 3 (11.1) 0 (0.0) 1 (3.7)	Employment status (n, %) Currently unemployed Working full or part time Retired Disabled Volunteering part or full time	1 (3.7) 14 (51.9) 8 (29.6) 4 (14.8) 0 (0.0)
Highest level of education (n, %) Less than high school High school diploma or GED Trade school/certificate program College degree (2-year associates) College degree (4- year bachelor) Graduate/professional school	0 (0.0) 6 (22.2) 4 (14.8) 5 (18.5) 7 (25.9) 5 (18.5)	Name of insurance (several responses possible) (n, %) Medicare Medicaid Medicare Part D Prescription drug Medicare medical savings account Medicare Advantage Employer provided/sponsored insu Privately arranged insurance Non-Medicare retired benefit Tricare/Veterans healthcare Other	13 (48.1) 2 (7.4) 7 (25.9) 0 (0.0) 6 (22.2) 6 (22.2) 3 (11.1) 0 (0.0) 0 (0.0) 6 (22.2)
Heart problems (n,%)	4 (14.8)	Difficulties in picking up things (n, %)	4 (14.8)
Health-related osteoporosis			
Osteoporosis diagnosis by a doctor (n, %)	27 (100.0)	Difficulties in daily life due to osteoporosis (n**, %)**	16 (59.3)
Fracture diagnosis by a doctor Yes (n, %) Fracture last year (n, %)	16 (59.3) 3 (11.1)	Location of fracture Wrist (n, %) Number of times (min-max) Age for first fracture (years) Spine (n, %) Number of times (min-max) Age for first fracture (years) Hip (n, %) Number of times (min-max) Age for first fracture	8 (29.6) 0-2 50-75 5 (18.5) 1-2 50-65 3 (11.1) 1 50-55
OP treatments prescribed by a doctor (n, %)	25 (92.6)	Number of OP treatments ever taken Min-max Median (P25-P75)	0-5 1.0 (1.0-3.0)
Type of treatment (several responses possible) (n, %) Weekly oral tablet Daily subcutaneous injection Monthly in-office injection 6-month subcutaneous injection Yearly intravenous injection Prescription received but never start medication Started but decided to stop Started but stopped (advice of family member/friend) Started but stopped (doctor’s orders) Other Do not know	15 (55.6) 4 (14.8) 0 (0.0) 8 (29.6) 4 (14.8) 1 (3.7) 5 (18.5) 1 (3.7) 2 (7.4) 3 (11.1) 0 (0.0)	How long take the treatment (n, %) <6 months Between 6 months and 2 years >2 years Do not currently take OP treatment	3 (11.1) 5 (18.5) 12 (44.4) 5 (18.5)
Osteoporosis of a biological parent diagnosis by a doctor (n, %)	18 (66.7)	Hip fracture of a biological parent (n, %) Age of father (min-max) Age of mother (min-max)	8 (29.6) 55-85 75
**Considerations for mode of administration**			
Preference of administration (n, %) By mouth By shot Other	23 (85.2) 3 (11.1) 1 (3.7)	Concern of treatment that requires frequent visit to doctor (COVID-19) (n, %)	11 (40.7)
Ever take an injectable treatment (n, %)	16 (59.3)	Ever inject yourself/family member (n,%) If yes, willing to do it again	9 (33.3) 9 (100.0)
If shot is prescribed by a doctor (n, %) Can give himself a shot Need help and have someone to ask Need help, but do not have anyone Would never take a shot	21 (77.8) 2 (7.4) 4 (14.8) 0 (0.0)	Concerned about side effects of treatments (n, %) Low level of concern Moderate level of concern High level of concern Very high level of concern	5 (18.5) 12 (44.4) 6 (22.2) 4 (14.8)
Patient perspective of needles (n, %) Needles have never bothered me Shots aren't pleasant, but they've never scared me away from needed treatment Afraid of needles and do not take shots	12 (44.4) 15 (55.6) 0 (0.0)	Ever miss taking medication (n, %) Often Sometimes Rarely Never Do not currently take drugs	0 (0.0) 1 (3.7) 13 (48.1) 8 (29.6) 5 (18.5)
Afraid to have blood drawn when going to doctor (n, %)	0 (0.0)	Ever stop treatment without telling a doctor (n, %)	11 (40.7)
Number of treatments prescribed (OP and not OP treatments) Min-max Median (P25-P75)	0-13 3.0 (1.0-5.0)	Cost of treatment (OP and not OP treatments) ($, min-max)	0-100

OP: Osteoporosis

#### Patient stated preferences for osteoporosis management

**Fig. 2.**
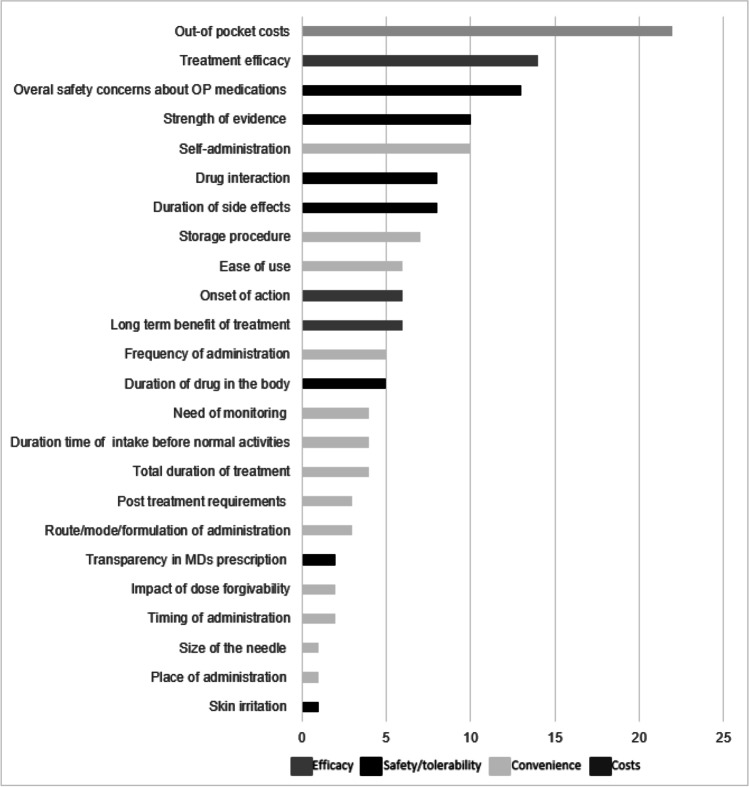
Number of times a treatment attribute was ranked in the top 7 of most important attributes (results from focus groups 1, 2 and 3 combined, n=27)

During focus groups, 10 additional treatment characteristics were generated by participants, which were not previously identified from the literature reviews. After consolidating insights from the focus groups and literature review and removing duplicate attributes, 24 treatment characteristics remained (Appendix D) with 3 attributes classified in the “Efficacy” domain, 7 in the “Safety” domain, 13 in the “Convenience” domain and 1 in the “Costs” domain. The number of times a treatment attribute was ranked in the top 7 most important attributes by participants is graphically represented in Fig. 2. Out-of-pocket costs, treatment efficacy, overall safety, strength of evidence, and self-administration were the five most important characteristics. The perspective of strength of evidence varied among individual patients and included whether the drug’s efficacy and safety were evaluated in large clinical trials, publication of the findings in peer-reviewed journals, FDA approval, and time since FDA approval together with reported safety signals. Four overarching themes of importance were discussed during focus groups including efficacy, safety, cost, and convenience.

##### Efficacy

Among the 27 participants, 29.6%, 40.7% and 51.8% ranked the efficacy attributes in their top 1, top 2 and top 3 important attributes, respectively. Most patients considered change in BMD T-score as the main indicator of treatment effectiveness. Additionally, the ability of the medication to build bones as well as the time to onset of action were considered important efficacy parameters.

##### Safety

29.6%, 70.4% and 85.2% of participants ranked the safety attributes in their top 1, top 2 and top 3 of most important attributes, respectively. The majority of patients understood the risk of side effects with osteoporosis treatments and expressed the importance of knowing what to expect before treatment initiation. For transient side effects, many agreed that minor side effects (i.e., skin irritation) which would resolve with time could be tolerated, except for one individual patient with a skin condition. Patients overall noted that more severe side effects that would endure after treatment discontinuation, and did not outweigh the benefits of therapy, would not be acceptable. The more severe the disease progression, saddled by increased pain or decreased functioning, the more willing patients were to accept the risk of transient side effects including moderate adverse events, as long as the medication would be effective in their fracture risk reduction.

##### Cost

The cost attribute was ranked by 29.6%, 40.7% and 51.8% participants in their top 1, top 2 and top 3 of most important attributes, respectively. Variation in ranking of cost attribute was driven by patients’ insurance coverage. While some patients expressed no issues with out-of-pocket cost, others expressed the need for copay assistance from pharmaceutical companies due to affordability issues. Women, older individuals, and those with a prior fracture were more likely to have cost in their top attribute of importance. Duration of treatment was also a consideration in selection of cost as an important attribute with concern about long-term affordability. For patients with Medicare coverage, the affordability perception was somewhat influenced by their experience with lower co-pays for medications requiring in-office injections compared to those requiring self-administration at home

##### Convenience

11.1%, 29.6% and 51.8% of participants ranked convenience in their top 1, top 2 and top 3 of most important attributes, respectively. Convenience covered a wide range of dimensions including self-administration, ease of use, route of administration, frequency of administration and storage requirements. Convenience was particularly important as it related to one’s ability to maintain everyday activities. Self-administration was one of the top 5 most important outcomes for patients (10/27 patients). Some patients felt that a treatment administered at home is more convenient than one requiring a visit to their physician.

Some differences in attribute ranking between exploratory subgroups were observed (Appendix E). Treatment efficacy, for example, was considered more important for women (60% of women selected this attribute among the 7 most important attributes versus 29% of men) whereas onset of action was considered more important for men (43% versus 15% of women). Safety/tolerability attributes were ranked high for patients older than 65 years (67% of older patients selected safety attributes in their top 7 versus 25% of younger patients) and for those without a fracture history (55% cared about duration of side effects versus 13% of patients with fracture history). Strength of clinical evidence was equally ranked between men and women, but it was considered more important for patients older than 65 years (53% versus 17% of younger patients ranked this attribute among their top 7 attributes), for those without a prior fracture (55% versus 25% of those with a prior fracture) and for patients without a profile of very high risk of fracture (53% versus 17% of those with a profile of very high risk of fracture). Finally, the self-administration characteristics offered with daily sMTS application were particularly important for women (45% of women selected this attribute among the 7 most important versus 14% of men) and for patients with a prior fracture (50% versus 18% of those without a prior fracture). Some differences in the importance ranking of attributes were also observed between the focus groups driven by the mix of participant characteristics in these groups.

#### Medication Route of Administration preferences

When questioned about the stated preference for route of administration, the majority of patients (96%) noted they would administer a daily sMTS if prescribed by their physician although none of them have previously experienced administering sMTS. The majority of patients rated the daily sMTS application as their first choice (84%), while 12.5% and 4.2% ranked in-office monthly injectable and an in-home daily injectable as first choice, respectively (Table 2). Stratified analysis of patients’ stated preference for route of administration demonstrated no significant difference between subgroups.

**Table 2.** Patient’s preference for route of administration

Questions	All (n=25)*
If doctor prescribed a daily skin patch to treat osteoporosis, would take it? Yes (n, %)	24 (96.0)
If doctor recommended a medication that will reduce risk of fracturing a bone by half and have a good safety profile, would take it? Yes (n, %)	24 (96.0)
Willing to accept a medication that does not work as well but costs less? Yes (n, %)	4 (16.0)
Think a cheaper medication is less likely to work than one that is more expensive? Yes (n, %)	5 (20.0)
Choice of administration	
Skin patch	
Rated as First choice (n, %) Rated as Second choice (n, %) Rated as Third choice (n, %)	21 (84.0) 4 (16.0) 0 (0.00)
Injection pen**	
Rated as First choice (n, %) Rated as Second choice (n, %) Rated as Third choice (n, %)	1 (4.2) 12 (50.0) 11 (45.8)
Injection from HCP**	
Rated as First choice (n, %) Rated as Second choice (n, %) Rated as Third choice (n, %)	3 (12.5) 8 (33.3) 13 (54.2)

*2 participants from the third focus group did not reply to these additional questions

**1 participant from the third focus group did not want to rank injections. The ranking for injection pen and injection from HCP is therefore solely based on 24 participants.

## Discussion

This study assessed US osteoporosis patient stated preference and relative importance of attributes when selecting between a novel mode of administration (sMTS) currently in development and the FDA-approved daily and monthly SC anabolic therapies. Twenty-four attributes were identified through two systematic literature reviews and three focus groups, and were categorized in “efficacy”, “safety/tolerability”, “convenience” and “costs” domains. Overall, out-of-pocket costs, treatment efficacy, safety, strength of evidence, and self-administration were considered most important by patients during online focus groups.

Only 14 out of the 24 (58.3%) characteristics identified in this study came from a literature review, highlighting the importance of mixed methods, involving literature review, expert consultation, and patient interviews for identification of all relevant treatment attributes. Some important treatment characteristics such as strength of evidence, onset of action, half-life of the drug, for example, emerged only from the focus group silent generation and not from literature review. One reason for this observation may be the fact that this is the very first study evaluating the stated preference for sMTS and SC-injections of an osteoanabolic agent. Since sMTS is still under investigation, no previous preference study using this mode of administration has been published. Data exist for other treatments administered via patch compared to injection where variations in patients’ acceptance of and satisfaction with treatment are reported [22–24]. Several studies highlighted a higher preference for transdermal patches versus oral medication both from patients and caregivers [25–28] in Alzheimer disease. One of the reasons for this preference may be the lack of stimulation of nerves associated with pain [31].

In addition to the assessment of stated preference for treatment attributes, this study also revealed acceptability and stated preference for sMTS. The majority of patients expressed willingness to apply sMTS daily if prescribed by their doctor. The results from this stated preference study are consistent with findings from preference studies in other disease areas where microneedle patches were generally preferred to SC-injections [24] in the majority of patients although the sMTS application is different and the study findings are limited to stated rather than observed preference. This mode of administration may have the potential to improve treatment initiation and persistence in patients who prefer not to use and injectable.

The findings reported here suggest consideration of patient perspective in osteoporosis treatment, consistent with earlier reports for patients with osteoporosis, and it is also aligned with the increasing importance of patient preference research in both clinical and policy decision making [29–31]. The FDA’s Patient Prioritization Endpoint initiative encourages collection and submission of data on patients’ preferences and unmet medical need since patients’ risk/benefit assessment of drugs may differ and should be considered during the review/approval process of new drugs [32]. The current study results highlight a higher risk tolerance for new therapies in patients with prior fractures and more progressed disease. An sMTS osteoanabolic treatment option may have the potential to improve treatment initiation and persistence in patients who may prefer not to use an injectable [5, 6]. Ideally, given variations in treatment preferences, patient/physician shared decision-making may lead to the best choice of treatment for an individual patient.

### Limitations

Interpretation of findings should be taken within the context of the study limitations. First, since the study focused on anabolic therapies only three modes of administration (i.e. daily sMTS application, daily SC-injection, and monthly SC-injection) were considered and other modes of administration including oral medications, once yearly intravenous injection or twice yearly sub-cutaneous injections for antiresorptive drugs were not evaluated.

Second, participants were asked to report their stated preference making choices over hypothetical scenarios; therefore, findings may not reflect observed preference on actual choices made in real life. Regardless, stated preference data are widely used since an understanding patient preference before an intervention is available may help in characterization of patients suitable for a particular treatment and shared decision making may improve adherence. Furthermore, a recent evaluation of patient perspective of actual sMTS in a usability study [13] suggests high acceptability, including global satisfaction and satisfaction with convenience at day 15 and 29 of treatment. Patients who administered sMTS ranked ease of use and convenience as top attributes of treatment.

Third, as with any focus group, the perspectives of the small sample size may not be representative of perspective of all patients at high risk for fracture. Aligned with the FDA guidance for patient preference studies, in order to increase generalizability of findings, we included a mix of patients representing the general population of patients at high risk for fracture [15]. We did not specifically assess “needle phobia” or recruit patients with needle phobia or needle aversion resulting in limited generalizability of findings to those patients. The study findings may have limited generalizability to patients with lower socioeconomic status since we included patients with access to internet and PC/iPhone. This approach was necessary since visual illustration of treatment options was key to this research. Furthermore, the online method is considered adequate by the FDA as it offers several advantages: participation is not limited to a geographical representation, participants can be in the comfort of their own homes, and potential for participants to see each other if using a web cam—thus, allowing the potential benefits of seeing facial expressions and no travel costs or focus group facility rental fees. Additionally, the online method was a safe alternative for research compared to face-to-face meeting due to COVID-19 pandemic restrictions. Despite a pre-meeting check with patients on connection and access to the online platform, there remained some technical challenges. Three of the thirty patients recruited were not able to join the online meeting despite testing prior to the focus group. The study type and inherent restricted sample size also prevented us to perform some specific subgroup analyses. For example, we were not able to assess whether patients with and without injection experience have different preferences. Previous studies have highlighted the potential impact of injection experience on patient preferences[33]. Patients with prior experience with self-injectable drugs are less likely to switch to another mode of administration, however, there are preference variations based on perspectives and interpretation of convenience. Further quantitative research would be interesting to compare preferences of patients previously exposed with injection with those who had no experience.

Fourth, we collected information on education and employment as proxies for socioeconomic status. While information on income level was not ascertained, data on other variables associated with drug utilization (i.e., insurance and co-pay) were collected. We were unable to assess the potential influence of income level specifically on the classification of attributes, including the importance of cost attribute.

Finally, the classification of attributes into domains may be prone to judgment bias. Some attributes could be classified in multiple domains from based on patient perspective. For example, dose forgivability could be considered a safety attribute by some and efficacy by others. For analyses purposes and to improve reliability, the research team approved the domain of inclusion of these attributes based on their clinical and research expertise.

## Conclusion

The current study findings suggest that efficacy, safety, out-of-pocket costs, and convenience are important attributes of treatment for US osteoporosis patients at high risk for fracture with a high stated preference for daily sMTS over daily or monthly SC-injections. The availability of sMTS may have the potential to improve treatment initiation and persistence with osteoanabolic therapy for US osteoporosis patients who would not accept injectable therapy. Observed preference study including perspective of patients who have experience with the given therapeutic options is needed. Further investigation of the trade-offs between treatment attributes including willingness to pay could further characterize patients who prefer one treatment over another.
